# Surface science at the PEARL beamline of the Swiss Light Source

**DOI:** 10.1107/S1600577516018646

**Published:** 2017-01-01

**Authors:** Matthias Muntwiler, Jun Zhang, Roland Stania, Fumihiko Matsui, Peter Oberta, Uwe Flechsig, Luc Patthey, Christoph Quitmann, Thilo Glatzel, Roland Widmer, Ernst Meyer, Thomas A. Jung, Philipp Aebi, Roman Fasel, Thomas Greber

**Affiliations:** a Paul Scherrer Institut, Villigen, Switzerland; b Universität Zürich, Zürich, Switzerland; c Nara Institute of Science and Technology (NAIST), Nara, Japan; d Institute of Physics, Academy of Sciences of the Czech Republic, Praha Czech Republic; e MAX IV Laboratory, Lund University, Lund, Sweden; f Universität Basel, Basel, Switzerland; g Swiss Federal Laboratories for Materials Science and Technology (Empa), Dübendorf, Switzerland; h Université de Fribourg, Fribourg, Switzerland

**Keywords:** X-ray photoelectron spectroscopy, X-ray photoelectron diffraction, scanning tunneling microscopy

## Abstract

The Photo-Emission and Atomic Resolution Laboratory (PEARL) is a new soft X-ray beamline and surface science laboratory at the Swiss Light Source. PEARL is dedicated to the structural characterization of local bonding geometry at surfaces and interfaces of novel materials, in particular of molecular adsorbates, nanostructured surfaces, and surfaces of complex materials. The main experimental techniques are soft X-ray photoelectron spectroscopy, photoelectron diffraction, and scanning tunneling microscopy (STM). Photoelectron diffraction in angle-scanned mode measures bonding angles of atoms near the emitter atom, and thus allows the orientation of small molecules on a substrate to be determined. In energy scanned mode it measures the distance between the emitter and neighboring atoms; for example, between adsorbate and substrate. STM provides complementary, real-space information, and is particularly useful for comparing the sample quality with reference measurements. In this article, the key features and measured performance data of the beamline and the experimental station are presented. As scientific examples, the adsorbate–substrate distance in hexagonal boron nitride on Ni(111), surface quantum well states in a metal-organic network of dicyano-anthracene on Cu(111), and circular dichroism in the photoelectron diffraction of Cu(111) are discussed.

## Abstract 2

The Photo-Emission and Atomic Resolution Laboratory is a new soft X-ray beamline at the Swiss Light Source for the study of surface structure using photoelectron diffraction and scanning tunneling microscopy.

## 1. Introduction   

### 1.1. Scientific case   

Surface science is an active, interdisciplinary field with applications in chemistry and physics such as heterogeneous catalysis, energy conversion, semiconductor and molecular electronics, spintronics and quantum magnetism. In these applications, chemical bonding, electronic charge transfer and magnetic interactions at interfaces play an important role, and many of the effects are intimately coupled to the atomic structure at the interface. Therefore, knowing the detailed structure is essential for the understanding of the underlying physics, and for the development and testing of theoretical calculations.

The Photo-Emission and Atomic Resolution Laboratory (PEARL) is a new soft X-ray beamline and surface science laboratory at the Swiss Light Source (SLS). It has been designed by a consortium of Swiss research groups active in surface science for the study of local atomic geometry at the surface of a wide range of novel organic and inorganic systems. Such systems include, for example, functional organic molecules (Fasel *et al.*, 1996 ▸; Muntwiler *et al.*, 2005 ▸; Pawlak *et al.*, 2012 ▸), supramolecular networks (Barth, 2007 ▸; Lobo-Checa *et al.*, 2009 ▸; Zhang *et al.*, 2014 ▸), molecular magnets (Scheybal *et al.*, 2005 ▸), chiral recognition (Fasel *et al.*, 2004 ▸; Greber *et al.*, 2006 ▸; Schillinger *et al.*, 2007 ▸), endohedral fullerenes (Treier *et al.*, 2009 ▸; Westerström *et al.*, 2012 ▸, 2014 ▸), ultrathin metal oxides (Jaouen *et al.*, 2015 ▸), surfaces of ferroelectrics (Despont *et al.*, 2006 ▸) and surface alloys (Corso *et al.*, 2010 ▸; Pawlak *et al.*, 2015 ▸).

Owing to the stringent requirements of ultra-high vacuum (UHV) in surface science experiments many of the mentioned samples have to be prepared *in situ* without breaking UHV conditions between preparation and measurement. It is therefore crucial to have standard analytical tools available which allow for a quick assessment of the sample quality before long-running measurements are made. Furthermore, it is important that complementary analytical methods can be applied on the same sample if the correlation between atomic structure and electronic or magnetic properties is investigated. At synchrotron-based facilities these requirements are particularly challenging since the focus on instrument development is often laid on one specific technique. At PEARL, the aspect of integrating a reasonably complete surface science laboratory at a synchrotron facility was the major design goal.

### 1.2. Technical case   

In surface science, charged particles, mostly electrons, are typically used as probes in a variety of experimental methods because they are particularly surface sensitive. At PEARL, the main analytical methods are soft X-ray photoelectron spectroscopy (XPS), photoelectron diffraction (XPD/PhD) as well as scanning tunneling microscopy (STM) and spectroscopy (STS). Since the instrumentation for XPD/PhD is mostly the same as for angle-resolved photoelectron spectroscopy (ARPES) in general, the facility also offers the spectroscopy of core-levels, valence bands (with limited resolution), Auger decays and resonant excitations. In the following, we will discuss mainly photoelectron diffraction and scanning tunneling microscopy.

Owing to their ease of use, scanning probe techniques have evolved to be popular methods for real-space imaging of surface structure and other surface properties at various length scales down to atomic resolution. However, vertical distance and angles between atoms are not easily accessible, and the contributions of the atomic and the electronic structure to a measured contrast are sometimes difficult to distinguish. Photoelectron diffraction is a technique to measure local atomic structure by exploiting the wave nature of photoelectrons and its diffractive properties at atomic potentials. Diffraction features appear as a variation of the photoelectron intensity as a function of emission angle and electron energy. By selecting a particular core-level photoemission peak, the technique is chemically selective, and due to a short mean free path it is sensitive to the topmost atomic layers. Photoelectron diffraction in angle-scanned mode (XPD) (Fadley, 1984 ▸; Osterwalder *et al.*, 1995 ▸; Fadley, 2010 ▸) is suitable for measuring bonding angles and the orientation of small molecules with respect to the substrate (Fasel *et al.*, 1996 ▸); while in energy scanned mode, where the acronym PhD is more commonly used, it is sensitive to the distance between neighboring atoms, for example, between adsorbed molecules and the substrate (Woodruff, 2007 ▸).

Energy-scanned PhD requires a photon source with smoothly tunable energy in the soft X-ray range. Tunable photon energy is also beneficial in many other cases. In photoelectron spectroscopy (XPS), the photoionization yield and the probing depth depend on photon energy, and the surface/bulk ratio can be adjusted. By tuning the photon energy to a particular X-ray absorption line, the photoemission cross section can be resonantly enhanced (Treier *et al.*, 2009 ▸), or a particular symmetry of the photoelectron wavefunction can be selected (Morscher *et al.*, 2011 ▸; Matsui *et al.*, 2015 ▸). Using the core-hole clock method with Raman-active Auger decays, ultrafast delocalization dynamics in the conduction band can be studied (Föhlisch *et al.*, 2005 ▸; Jaouen *et al.*, 2015 ▸). In angle-scanned XPD the ratio between forward and backward scattering can be tuned.

**Figure 1:**
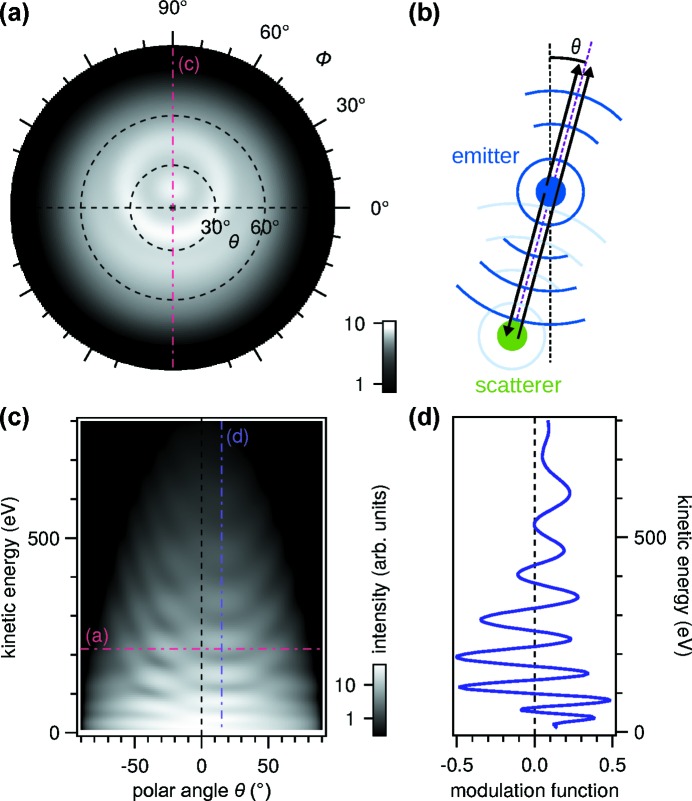
Calculated photoelectron diffraction *versus* angle and energy for a diatomic system consisting of a nitrogen atom as emitter and nickel as scatterer. (*a*) Hemispherical angle-distribution of the photoelectron intensity in stereographic projection. The gray scale is logarithmic. (*b*) Schematic electron scattering geometry. The nearest-neighbor direction is tilted by 15° with respect to normal emission. The azimuthal angle is 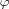 = 90°. (*c*) Photoelectron intensity as a function of kinetic energy and polar angle θ. Corresponding section lines to panels (*a*) and (*d*) are indicated. (*d*) One-dimensional modulation function extracted from panel (*c*) along the vertical line at 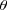 = 15°. A detailed description is given in the text.

Fig. 1 ▸ illustrates the basic measurement principle of XPD in the hypothetical example of two atoms. Let us assume a nitrogen atom is bonded to a nickel atom below, and the connecting line of the two atoms is tilted by 15° with respect to the reference axis (the sample normal in an actual system). This diatomic cluster is a reduced version of the h-BN/Ni system to be discussed in the *Scientific highlights* section[Sec sec4] below. At a fixed kinetic energy of 215 eV, the intensity of the N 1*s* XPS peak exhibits the angular distribution shown in panel (*a*), as calculated using the *EDAC* electron diffraction code (García de Abajo *et al.*, 2001 ▸). The angular distribution of the intensity is displayed in a stereographic projection: the polar coordinates of the unit hemisphere 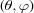 are mapped to planar Cartesian coordinates according to 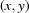 = 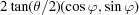. The wavefunction of the photoelectron emanating from the emitter atom is scattered at the neighboring atom, and the direct and scattered waves interfere in the detector. The results are characteristic, circular interference fringes around the nearest-neighbor direction. Such features have been used in experiments to locate the adsorption site of atoms on metal surfaces, for example, in the case of O/Rh(111) (Wider *et al.*, 1998 ▸).

If we cut the angular distribution of the photoelectron intensity along the 90° azimuth and expand the calculation in kinetic energy, we obtain the pattern shown in Fig. 1(*c*) ▸. The interference fringes appear again centered on the angle of the connecting line between the atoms. The frequency of the fringes is given by the distance between the two atoms, and the energy-dependent wavelength of the photoelectron. In the plot (note the logarithmic gray scale) it is obvious that the diffraction features typically correspond to a small variation of intensity on a strong but slowly varying background. Theoretically, this is due to the angular dependence of the photoemission cross section and the scattering factors. In the experiment, additional factors such as photon flux, sample orientation and detection efficiency may contribute, and are often difficult to separate from the diffraction signal. The relevant diffraction features are extracted by calculating the modulation function
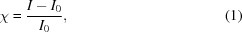
where 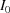 is a smooth function from a non-parametric fit of the data, such as a cubic spline or locally weighted linear regression (Woodruff, 2007 ▸). The modulation function of the present data set near 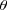 = 15° is shown in Fig. 1(*d*) ▸. Experiment and theory can be compared quantitatively by calculating the Pendry *R*-factor of the modulation functions (Woodruff, 2007 ▸),
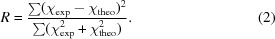
Conventionally, PhD scans were measured as one-dimensional line scans similar to the profile in Fig. 1(*d*) ▸. However, modern angle-dispersive analysers make it easy to measure multiple directions in parallel as in Fig. 1(*c*) ▸. This has two advantages over line scans. First, the precise emission angle can be determined from the same dataset as the distance between emitter and scatterer. Second, if the data contain diffraction features from multiple scattering configurations, correspondingly, more structural parameters can be determined at once.

## 2. Technical setup   

### 2.1. Beamline optics   

The specifications for the X-ray optics are based on the scientific and technical case described above. The details of the optical design have been discussed in a previous article (Oberta *et al.*, 2011 ▸). Essentially, the beamline covers the photon energy range from 60 to 2000 eV. It is optimized for high photon flux in the range between 500 and 1000 eV where most photoelectron diffraction measurements of the lighter elements take place. Higher photon energies give access to resonant excitation of 4*f* levels in rare earths, and the low end allows for basic spectroscopy of the valence region. The key figures are summarized in Table 1 ▸.

**Figure 2:**
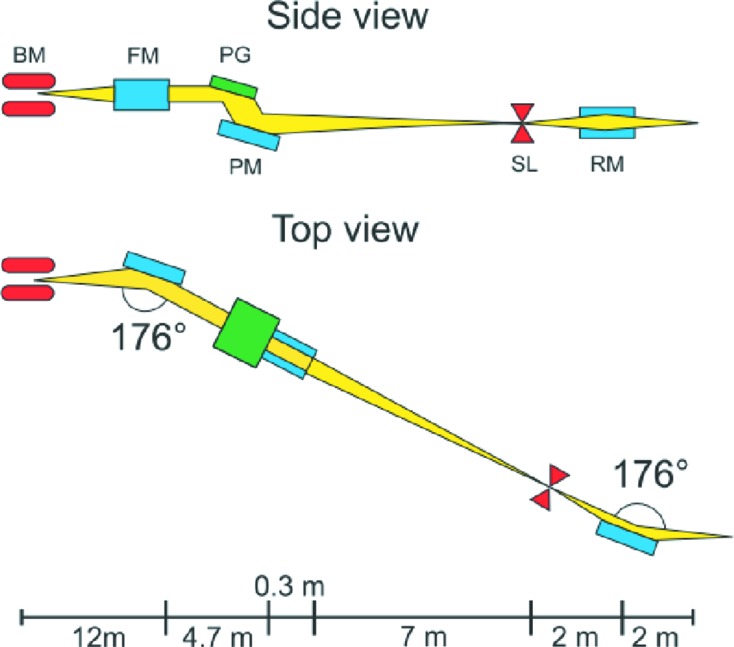
Schematic layout of the beamline, showing the optical path of the X-rays from the bending magnet to the endstation. The principal optical elements are: bending magnet (BM), focusing mirror (FM), plane grating (PG), plane mirror (PM), exit slit (SL), refocusing mirror (RM). Reprinted from Oberta *et al.* (2011 ▸) with permission from Elsevier.

The beamline is installed at a 1.4 T bending magnet which delivers a smooth photon spectrum with a critical energy of 5 keV. The main polarization mode of the bending magnet is linear horizontal. By tilting the trajectory of the stored electron beam, the polarization can be switched to elliptical. The optical layout is based on a plane-grating monochromator (Petersen *et al.*, 1995 ▸) operating in non-collimated light and negative diffraction order, as shown schematically in Fig. 2 ▸. This scheme provides a good compromise between high photon flux and high energy resolution. In the optimum energy range, it allows to distinguish chemically shifted core levels or spin multiplets of the order of 0.1 to 0.5 eV. For systems where high resolution is not needed, it is possible to trade resolution for flux. Compared with other soft X-ray beamlines at the SLS operating in collimated light (Strocov *et al.*, 2010 ▸; Piamonteze *et al.*, 2012 ▸), the number of reflecting surfaces is reduced by one to save photon flux. As a drawback, the fixed-focus condition is set by design and cannot be modified during operation, giving the user less control over the harmonics in the spectrum. Using two selectable diffraction gratings (600 and 1200 lines mm^−1^), photon energy is smoothly tunable in two overlapping energy ranges (60–1100 and 200–2000 eV, respectively).

Although the signal-to-noise ratio in photoelectron diffraction can benefit strongly from high photon flux, many samples, particularly organic molecules, are susceptible to radiation damage if the flux is too high. To mitigate the problem, it is not sufficient to reduce the photon flux because that reduction would have to be compensated by increased exposure time. Rather, high electron yield with high conversion and detection efficiency is required. For the one part this is achieved with tunable photon energy, as the photoionization cross section can vary by orders of magnitude over the energy range of soft X-rays. For the other part, the photon flux can be spread over a larger area of the sample, thereby reducing the flux density, while the entire illuminated sample area is seen by the detector. At PEARL, the refocusing mirror unit can be switched to produce either a focused or a defocused beam on the sample.

### 2.2. Experimental station   

**Figure 3:**
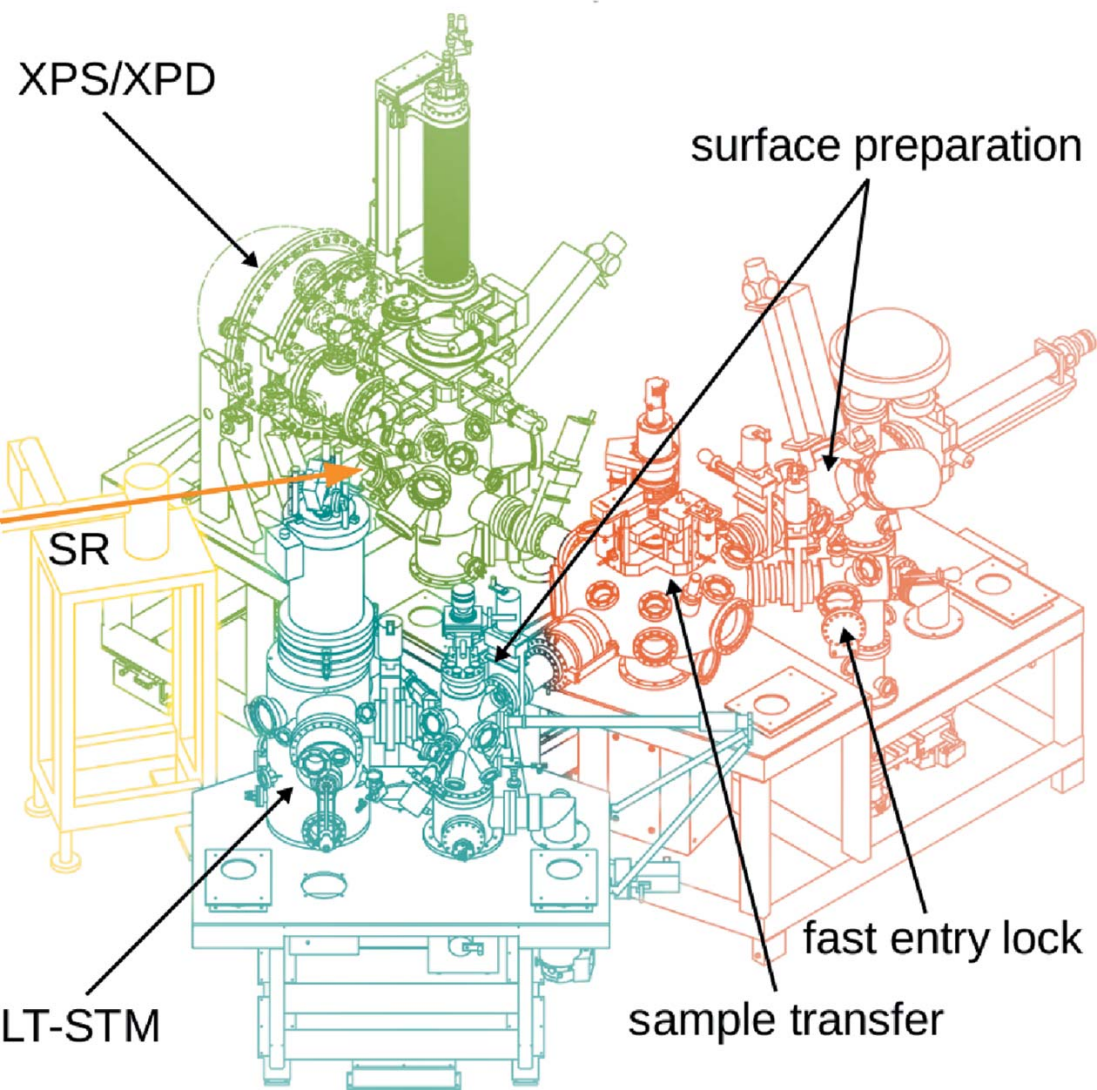
Conceptual rendering of the endstation. The three substations for angle-resolved photoelectron spectroscopy and diffraction (XPS/XPD, green), scanning tunneling microscopy (LT-STM, blue) and surface preparation (red) are connected to a central, rotary sample transfer under UHV. The synchrotron radiation (SR) enters the XPS/XPD station along the path marked by an arrow. The drawing does not accurately represent the installation status of auxiliary devices.

The experimental station is divided into three sub-systems (Fig. 3 ▸): one (attached to the beamline) for the photoemission measurements, one for scanning tunneling microscopy and one for surface preparation. All processes and measurements take place in UHV at a base pressure below 2 × 10^−10^ mbar. The sub-systems are connected by a reliable *in situ* sample transfer system. Though the system operates at room temperature, the transfer time between measurement positions is short enough to prevent cold samples (below 100 K initially) from heating up above 200 K. Samples and organic powders for evaporation are introduced from ambient or a UHV suitcase *via* a fast-entry lock.

The sample preparation system provides standard surface science techniques for preparation (ion bombardment, annealing by radiative heating) and characterization [low energy electron diffraction (LEED), Auger electron spectroscopy, residual gas analysis]. A sixfold array of molecular beam evaporators with *in situ* exchangeable quartz crucibles for materials which sublimate below 900 K is available, as well as gated ports for user-supplied evaporators. A high-temperature annealing stage (1500 K) is under construction. For full specifications, see Table 2 ▸.

The low-temperature STM (Omicron Nanotechnology GmbH, Table 3 ▸) provides real-space sample characterization down to atomic resolution. Standard topography mode allows for quick assessment of the surface quality and reference to measurements at the user’s home laboratory, 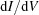 spectroscopy and mapping can be used to measure the local density of states near the Fermi level. Thanks to careful damping inside and outside the chamber, the STM has proven insusceptible to vibrations and acoustic noise from the synchrotron environment.

**Figure 4:**
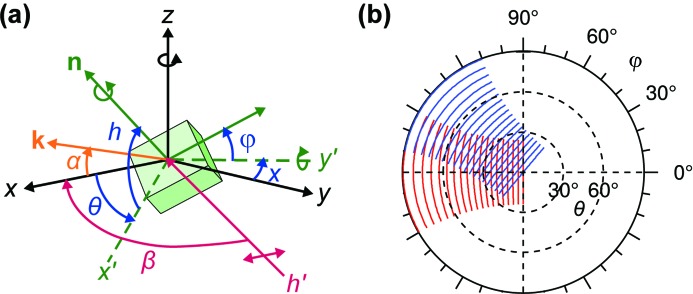
(*a*) Measurement geometry in the ARPES chamber. The coordinate axes *x*, *y* and *z* are fixed in the laboratory frame of reference. Photoelectrons are detected in the *xz* plane under the acceptance angle −30° 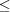 α 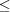 +30° centered on the *x* axis. The synchrotron beam enters at an angle 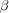 = 60° with respect to the *x* axis, the polarization vector of horizontal light is in the *xy* plane. The sample can be moved in the *x*, *y* and *z* directions, and rotated about the *z* (polar angle θ) and *y*′ (tilt angle ψ) axes, as well as about the surface normal 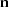 (azimuthal angle φ). (*b*) Scanning scheme of angle-scanned photoelectron diffraction in the spherical coordinate system in stereographic projection. A full scan of emission angles in the hemisphere is a combination of polar (θ) and azimuthal (φ) scans. Each of the curved lines in the plot corresponds to the angle range detected in one shot. For clarity, only a few angles are shown.

The photoemission station is designed as a state-of-the-art ARPES facility with a ‘Carving 2.0’ six-axis manipulator designed by PSI and Amsterdam University, and a Scienta EW4000 hemispherical electron analyser with two-dimensional detection. The specifications are summarized in Table 4 ▸. The measurement geometry is illustrated in Fig. 4(*a*) ▸. The entrance lens stack of the analyser is at a fixed angle 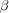 = 60° with respect to the incoming synchrotron light. The entrance slit of the analyser is oriented vertically (parallel to the main axis of rotation). In this orientation, the symmetry of the differential photoemission cross section with respect to the light polarization allows for a homogeneous illumination of the detector.

The primary rotation axis is the polar rotation θ about the *z* axis. The secondary rotation axes are the tilt ψ about the *y*′ axis, and the azimuthal rotation φ about the surface normal 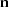 of the sample. The new version 2.0 of the Carving manipulator features an improved bearing concept of the primary rotation to reduce the sphere of confusion: three-dimensional mechanical test measurements after assembly show that, under polar rotation θ, the sample moves by less than 25 µm in the scattering 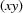 plane and less than 65 µm along the *z* axis. For the secondary rotation axes ψ and φ, the displacement is less than 25 µm. Such high mechanical precision is essential for angle-scanned measurements due to the small beam size and the small focal depth of the analyser, either of which is of the order of 100 µm. To take advantage of the high precision, however, the sample must be mounted with the same precision on the sample plate so that the surface is aligned with the rotation center of the manipulator. Usually, this requires a precise optical survey of the shape of the specimen and the manufacturing of a sample holder that is tailored to the specific shape.

The sample can be cooled down to 35 K using liquid helium (LHe). The actual sample temperature was confirmed by adsorption and desorption of argon on a Cu(111) surface, compared with literature values of the desorption temperature (Berthold *et al.*, 2004 ▸; Meyer *et al.*, 2008 ▸). Due to the particular design of the Carving manipulator, thermal contraction of the primary axis due to cryogenic cooling is negligible.

The EW4000 electron analyser contains a two-dimensional multi-channel plate detector where one axis corresponds to the kinetic energy of the electron and the other axis to the emission angle α. The nominal acceptance angle of this detector is 60°. In practice, transmission and matrix element effects limit the useful range to about 50°. Combining the manipulator and detector angles, photoelectron counts are collected as a function of the four angles θ, ψ, φ and α. However, in presentation graphs, angle-scanned photoelectron diffraction data are typically displayed in the spherical coordinate system 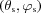 of the sample as in Fig. 1(*a*) ▸. Assuming that the angular dependence of the matrix element can be neglected (*e.g.* by normalization), instrument coordinates are mapped to sample coordinates by applying a series of rotations to the Cartesian vector 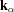 = 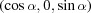 which marks the detection angle in the laboratory frame of reference (Greif *et al.*, 2014 ▸). In the sample frame, the emission vector 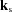 becomes, thus
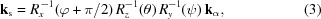
where 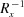, 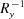 and 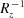 denote the inverse rotation matrices about the coordinate axes *x*, *y* and *z*, respectively. 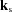 can then be mapped to spherical coordinates in the canonical way taking the *x* axis as the surface normal. Fig. 4(*b*) ▸ shows the lines accepted by the analyser for a number of manipulator positions and how they map to the sample frame according to equation (3). On the unit hemisphere, each of the lines corresponds to an arc of a great circle. In the stereographic projection, it appears curved with a θ-dependent curvature. A full hemispherical diffractogram can be measured by combined 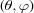 scans with a step size of 1° or 2° for θ, and between 15° and 50° for φ in typically 6 to 24 h, depending on the signal and desired amount of oversampling.

## 3. Measured performance   

### 3.1. Photon flux   

**Figure 5:**
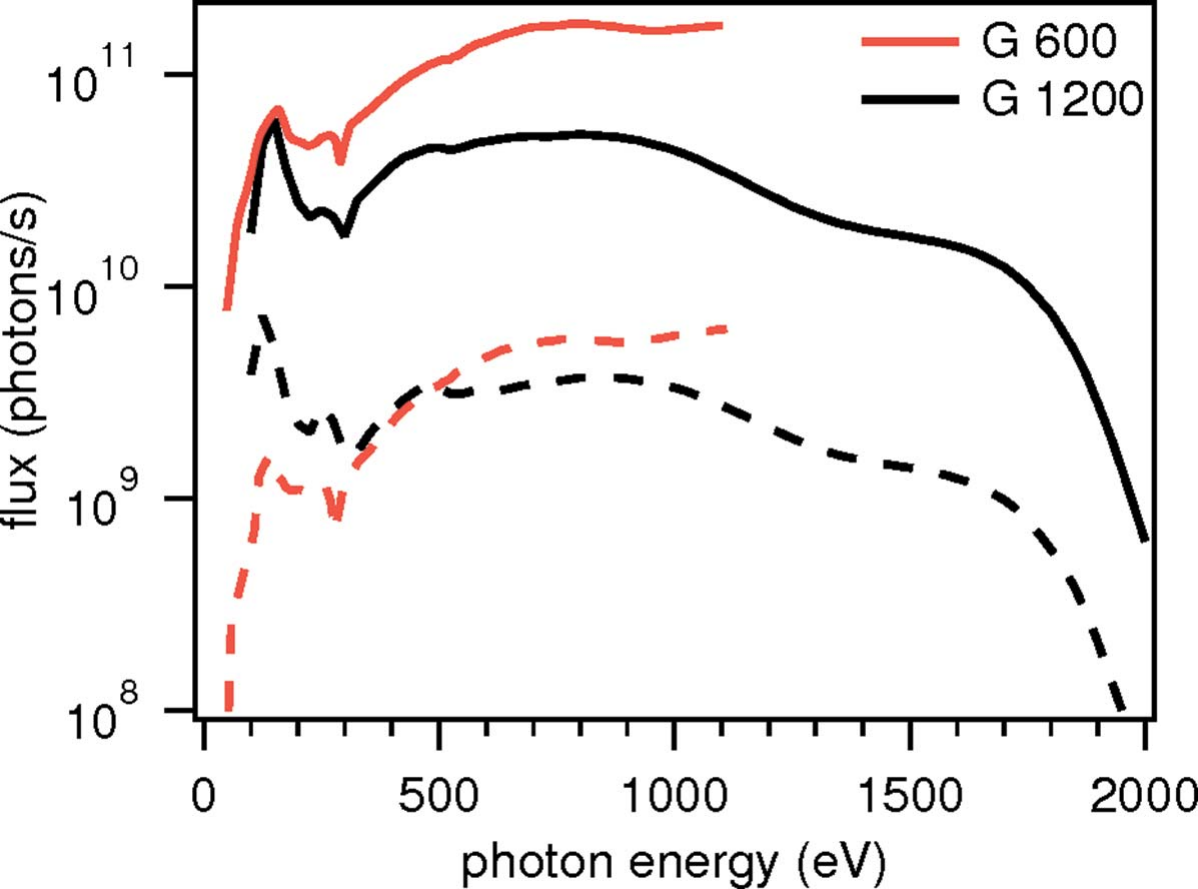
Photon flux after the refocusing mirror measured on a calibrated silicon photodiode for the 600 and 1200 lines mm^−1^ diffraction gratings. The solid and broken lines were measured for maximum (720 µrad × 1200 µrad) and minimum (120 µrad × 120 µrad) front-end aperture, respectively. The exit slit size is 100 µm.

The photon flux is measured by a calibrated silicon diode after the refocusing mirror, Fig. 5 ▸. The two laminar diffraction gratings cover an energy range from 60 eV up to 2000 ▸ eV with an overlapping region between 100 and 1000 eV. The 600 lines mm^−1^ grating is optimized for high photon flux, whereas the 1200 lines mm^−1^ grating is required for photon energies above 1000 eV, or for better energy resolution below 1000 eV. The dashed and solid lines mark the practical lower and upper limits which can be set by the front-end aperture, respectively, at a typical exit slit aperture of 100 µm. The upper limit is given by the physical size of the focusing mirror. The results are summarized in Table 5 ▸.

### 3.2. Energy resolution   

The energy resolution in photoelectron spectroscopy is limited by the beamline optics and the electron analyser. In this section, we first demonstrate the ultimate resolution of the optics by measuring gas phase X-ray absorption spectra of nitrogen. Second, we discuss the energy resolution of the complete system derived from photoelectron spectra under typical measurement conditions. In most practical cases, it is necessary to find a compromise between energy resolution and count rate by opening the apertures of the beamline and the analyser.

**Figure 6:**
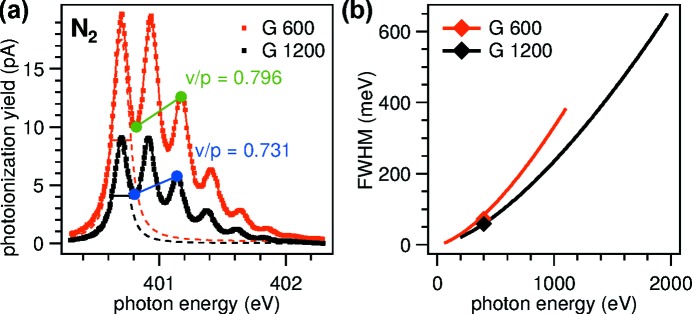
(*a*) Total yield absorption spectra of nitrogen measured with monochromatic light from the 600 lines mm^−1^ grating (G 600) and the 1200 lines mm^−1^ grating (G 1200), at an exit slit setting of 50 µm. Solid lines are least-squares fits of Voigt functions as described in the text, dashed lines show the decomposed lowest-energy peak. The intensity ratio between the first valley and third peak (v/p) is indicated. (*b*) Calculated energy resolution of the beamline optics at the aperture settings of the nitrogen spectra. Experimental values from nitrogen XAS are marked.

Total ion yield gas phase spectra are measured in a gas cell installed after the exit slit of the monochromator. The apertures are set at the lowest practical values of 1 mm × 1 mm for the front-end, corresponding to an acceptance angle of (120 µrad)^2^, and 50 µm for the exit slit. The measured N_2_ 1*s*–π* spectra are plotted in Fig. 6 ▸. The ratio of total yield between the first valley at 400.8 eV and the third peak at 401.2 eV is a sensitive measure of the overall energy resolution where lower values indicate better energy resolution (Chen & Sette, 1989 ▸). The advantage of the valley-to-peak (v/p) ratio over curve fitting is that it is independent of the calibration of the energy scale and less susceptible to correlations between fit parameters. For a quantitative measure, the spectrum is modeled with the sum of seven Voigt profiles and v/p is compared with the measurement. The basic parameters for the model spectrum, the natural line width of 113 meV FWHM and peak positions, are taken from the literature (Kato *et al.*, 2007 ▸). The resulting values for the resolving power are 5550 and 6860 for the 600 lines mm^−1^ and 1200 lines mm^−1^ gratings, respectively, very close to the corresponding values 5500 and 7000 from the design calculations (Oberta *et al.*, 2011 ▸). Benchmark values are summarized in Table 5 ▸. Detailed results and an additional discussion of curve fits are given in the supporting information.

**Figure 7:**
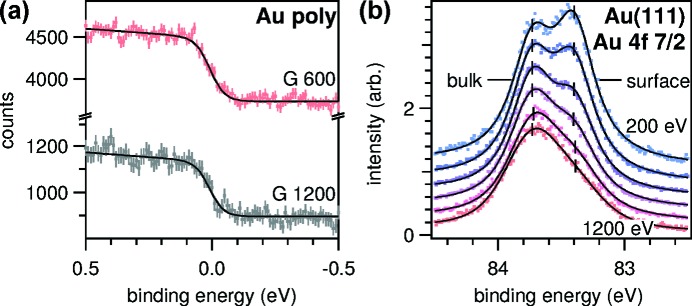
(*a*) High-resolution XPS spectra at the Fermi edge of polycrystalline gold measured at 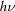 = 400 eV. Dots are electron counts integrated over the 60° acceptance angle of the analyser with error bars estimated according to the Poisson distribution. Lines are curve fits of a Fermi function assuming, to first order, a linear increase of the density of states below 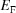. (*b*) High-resolution XPS spectra of the Au 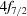 peak of a single-crystal Au(111) surface measured at a series of photon energies between 200 and 1200 eV. Solid lines are curve fits of two Voigt profiles. The weighted mean of the binding energy of the bulk peak is 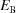 = (83.73 ± 0.01) eV, and the surface core-level shift is (0.329 ± 0.001) eV. The spectra are normalized to the area of the bulk peak and vertically offset for clarity.

To check the energy resolution of the complete system we measure the width of the Fermi edge of a polycrystalline gold sample with XPS. The spectra in Fig. 7 ▸ are taken at essentially the same beamline settings as the nitrogen absorption spectra except that the front-end aperture is widened to (240 µrad)^2^ to increase the count rate. The effect of the wider aperture on the energy resolution is less than 5% as confirmed in separate XAS measurements of nitrogen. The width of the Fermi edge contains two components, the intrinsic thermal broadening of the electron distribution in the material and the instrumental broadening by the analyser and the beamline. To first order, these effects add up quadratically as discussed in the supporting information (Kreutz *et al.*, 1998 ▸). To reduce the first contribution as much as possible, we cool the sample to 40 K. The spectra can be fit with a Fermi–Dirac distribution at 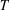 = (341 ± 40) K and 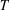 = (325 ± 62) K, which amounts to a total instrumental broadening of 103 and 98 meV, respectively. Given the resolution of the X-ray optics discussed before, the analyser resolution is estimated to be (76 ± 17) meV at the selected entrance slit (0.2 mm) and pass energy (50 eV). Though the resolution of the analyser could be improved by lowering the pass energy, the low count rate due to the very low photoemission cross section of the valence band in the soft X-ray regime did not allow so as the acquisition of each displayed spectrum took about 12 h. On the other hand, the spectrum of an intense peak such as the 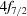 core level resolving the surface core-level shift as in Fig. 7(*b*) ▸ can be acquired with the same high-resolution settings in less than 10 min. These measurements demonstrate that the beamline is capable of resolving chemical shifts of core-levels of the order of 100 meV. However, at very high resolution and for low cross-section transitions, the count rate is limited.

### 3.3. Spot size   

**Figure 8:**
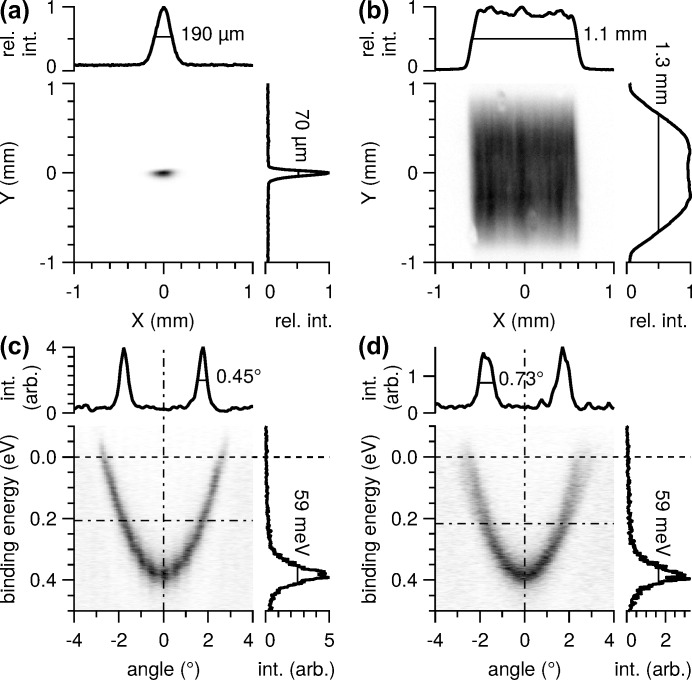
Small spot (*a*, *c*) *versus* large spot (*b*, *d*) setting of the refocusing mirror. (*a*, *b*) Distribution of X-ray flux on a scintillator plate at normal incidence at the designated sample position. (*c*, *d*) ARPES measurements of the Shockley state of the Cu(111) surface at 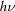 = 70 eV. The measurements were taken in fixed-energy mode at a pass energy of 10 eV and integrated over 10 min. The line graphs show the integrals over one dimension (*a*, *b*), or the profiles along the dash-dotted lines (*c*, *d*), respectively. Full width at half-maximum is indicated.

The spot size on the sample is a result of the size of the electron beam in the bending magnet, the optical magnification, aberrations and manufacturing tolerances. These effects sum up to a theoretical minimum spot size of 170 µm × 73 µm on the sample as predicted by ray-tracing calculations (Oberta *et al.*, 2011 ▸). Experimentally, the beam profile is measured on a scintillator plate at the nominal focus position of the refocusing mirror, *cf.* Fig. 8 ▸. The results for the small spot geometry, panel (*a*), agree very well with the calculations. The minimum FWHM spot size observed is 190 µm × 70 µm at a photon energy of 1000 eV and a vertical exit slit aperture of 100 µm. The small spot is almost independent of the front-end aperture and the photon energy. Its horizontal width increases slightly towards lower photon energy (230 µm at *h*ν = 400 eV).

The large spot setting of the refocusing mirror is designed to produce a convergent beam with an image distance of 5.7 m in the meridional (horizontal) plane, and a divergent beam with an image distance of −28.8 m in the sagittal plane. The observed spot size depends significantly on the front-end acceptance, varying from 180 µm × 160 µm at the smallest aperture (not shown) to 1.1 mm × 1.3 mm at the maximum aperture [panel (*b*)].

Since the electron optics of the EW4000 analyser is optimized for a small spot of 100 µm, it is interesting to check the effect of the spot size on the angle and energy resolution of the analyser. For this, we measure the Shockley surface state on Cu(111) at a photon energy of 70 eV [Figs. 8(*c*) and 8(*d*) ▸]. High-resolution measurements of this system are available in the literature (Reinert *et al.*, 2001 ▸). It is obvious that in the large spot configuration the angle distribution is broader than in the small spot configuration where the peak width approaches the nominal angle resolution of the analyser which is limited by the entrance mesh of the wide-angle lens. The influence of the spot size on the energy resolution is not obvious. In either case, the line width at the apex of the dispersion curve is 59 meV, limited by the instrumental energy resolution used in these measurements. We also find that the electron count rate is about 26% lower for the large spot while the total yield (71 pA) does not change. Because this loss of electron counts has to be compensated by longer integration time, the advantage of the large spot (longer protection against radiation damage due to lower flux density), is reduced from the original ratio of beam size to about a factor 80.

## 4. Scientific highlights   

### 4.1. Measuring adsorbate–substrate distance in boron nitride   

**Figure 9:**
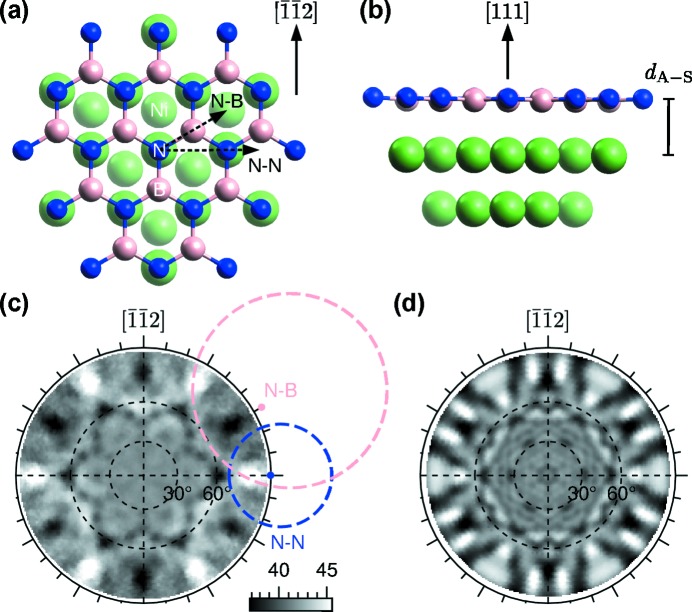
Angle-resolved photoelectron diffraction of the N 1*s* peak of h-BN/Ni(111) at 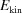 = 399 eV. (*a*, *b*) Cluster of atoms used in calculations in (*a*) top view and (*b*) side view. (*c*) Processed experimental data. The raw data consist of short XPS spectra (11 s each) measured at 2148 angular settings distributed over the hemisphere (15° steps in φ and 1° steps in θ). The hemispherical diffractogram is assembled from 50° wide detector images, normalized, and three-fold averaged as described in the text and the supporting information. The diffraction cones from scattering along the N–N and N–B nearest-neighbor directions [corresponding to the dashed arrows in panel (*a*)] are marked by dashed circles. (*d*) Calculated diffractogram from the best-fit structural model. The pattern is twofold averaged to match the symmetry of the measurement.

As a scientific example, we show angle- and energy-scanned photoelectron diffraction of hexagonal boron nitride (h-BN) on a Ni(111) surface. h-BN is a well known atomically thin insulating layer that can be used to chemically and electronically decouple molecular adsorbates from the underlying metal (Muntwiler *et al.*, 2005 ▸). The atomic structure of h-BN/Ni(111) has been studied by low-energy electron diffraction, angle-resolved photoelectron diffraction, scanning tunneling microscopy, and density functional theory in the past (Gamou *et al.*, 1997 ▸; Auwärter *et al.*, 1999 ▸; Muntwiler *et al.*, 2001 ▸; Grad *et al.*, 2003 ▸). h-BN forms a commensurate 1 × 1 overlayer with a nitrogen atom at the top site and a boron atom at the f.c.c. hollow site, *cf*. Figs. 9(*a*) and 9(*b*) ▸. The layer is slightly corrugated due to a lattice mismatch and different bonding of nitrogen and boron to the substrate. The distance between the h-BN layer and the substrate, *d*_A–S_, was studied by LEED previously and reported as 2.22 Å (Gamou *et al.*, 1997 ▸). A later angle-resolved XPD study reported a different value of 1.95 Å (Muntwiler *et al.*, 2001 ▸). Here, we look for an independent result using energy-scanned PhD in the backscattering configuration.

Angle-resolved XPD has the advantage that directions of atomic bonds can often be identified rather easily in a stereographic mapping of the photoelectron intensity without the need for a calculation. Such a map also helps to find the correct manipulator position with respect to specific diffraction features or bond directions for subsequent spectroscopy or PhD measurements. The diffraction pattern of the N 1*s* peak of h-BN in Fig. 9(*c*) ▸ is assembled from XPS spectra measured at 2148 angular settings according to the procedure described in the supporting information. The polar angle dependence of the data is removed by normalization (see below), and a threefold average is applied according to the symmetry of the substrate. The diffraction pattern shows notable rings at θ > 60° that are centered on the nitrogen–boron (N–B) and the nitrogen–nitrogen (N–N) nearest-neighbor directions. In contrast to earlier published data (Auwärter *et al.*, 1999 ▸), the pattern in Fig. 9(*c*) ▸ is sixfold symmetric due to the presence of two domains rotated by 180° with respect to each other. It is known that, in addition to the most stable N-top, B-f.c.c. adsorption configuration, an N-top, B-h.c.p. configuration with a slightly lower binding energy can grow depending on a subtle difference in the quality of the substrate (Auwärter *et al.*, 2003 ▸; Grad *et al.*, 2003 ▸). The Ni crystal used in the present experiment was newly procured, and had not undergone the same number of cleaning steps as the one in the previous studies. Panel (*d*) shows the corresponding simulation using the *EDAC* multiple-scattering code (García de Abajo *et al.*, 2001 ▸). The cluster in Figs. 9(*a*) and 9(*b*) ▸, showing only the most stable structure, is based on the optimized structural parameters discussed below. Qualitatively, measurement and calculation show the same diffraction features. However, a shift of features at higher polar angles indicates that the refraction at the surface may not be accurately described in the model.

To determine the adsorbate–substrate distance *d*_A–S_ between the N and top-layer Ni atoms, the PhD intensity modulation of the N 1*s* peak is measured as a function of electron energy and simulated numerically using the *EDAC* code. In the simulations, seven structural and non-structural parameters are optimized using a *particle swarm* global search algorithm (Duncan *et al.*, 2012 ▸) which minimizes the Pendry *R*-factor, equation (2). The optimized parameters are the adsorbate–substrate distance *d*_A–S_, the corrugation of h-BN, the possibly relaxed distance between the top two nickel layers, the size of the cluster, the position of the refractive surface above the top layer, and the amplitude of the modulation function.

**Figure 10:**
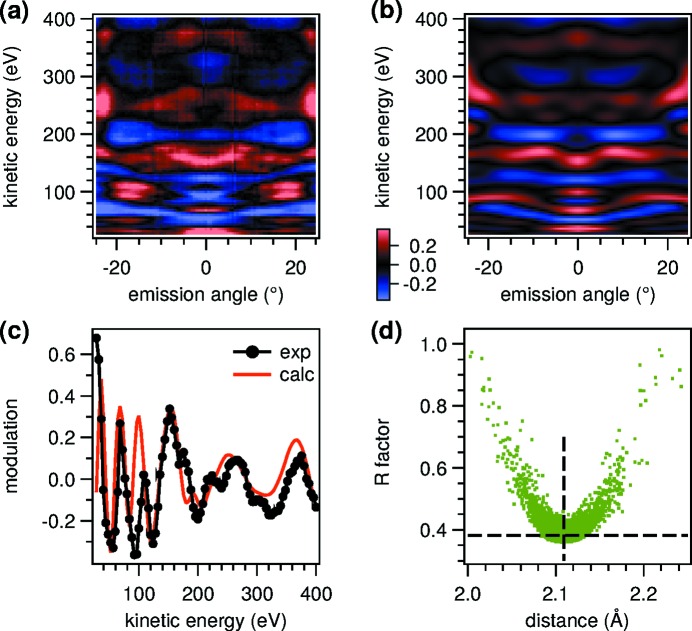
Energy-scanned photoelectron diffraction of the N 1*s* peak of h-BN/Ni(111) measured in backscattering geometry. (*a*) Two-dimensional experimental modulation function. The horizontal scale is the polar emission angle in the 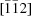 azimuth. (*b*) Calculated best fit modulation function. (*c*) One-dimensional modulation functions extracted from the experimental and calculated two-dimensional datasets at normal emission. (*d*) *R*-factor distribution *versus* adsorbate–substrate distance.

The measured modulation function normalized according to equation (1) is shown in Fig. 10 ▸, panels (*a*) and (*c*). Panel (*a*) shows the full two-dimensional dataset 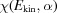 while panel (*c*) shows a line profile 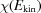 integrated over −2.5°< α < 2.5°. The corresponding simulations of the optimized model structure are shown in panels (*b*) and (*c*). Panel (*d*) shows the *R*-factor results from over 10000 calculated configurations as a function of *d*_A–S_, the main parameter of interest. It shows a strong dependence on the adsorption parameter where the minimum 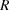 = 0.36 designates the best-fit value, and the width of the distribution can be used to estimate the uncertainty according to Booth *et al.* (1997 ▸). The result is *d*_A–S_ = (2.11 ± 0.02) Å.

Since the raw data of Fig. 10(*a*) ▸ were measured with a two-dimensional detector, we have carried out the optimization procedure for the two-dimensional and one-dimensional datasets separately. As can be seen in panels (*a*) and (*b*), the agreement between the calculation and the experiment is not reached in every detail, and the absolute values of the *R*-factor are correspondingly large. Nevertheless, the locations of the minima of *d*_A–S_ are compatible in both cases. The advantage of a two-dimensional dataset is that multiple angles are measured at the same time. In the present case, this allows for a (coarse) optimization of other parameters such as the relaxed distance between the top two nickel layers (1.99 ± 0.04) Å, which is not possible from the normal emission measurement alone because the back-scattering directions are off-normal.

**Figure 11:**
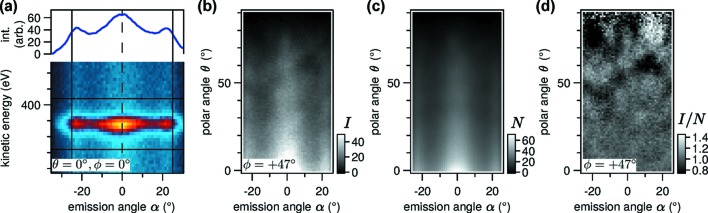
Normalization procedure of angle-scanned XPD data of h-BN/Ni(111). (*a*) Measured photoelectron intensity of the N 1*s* peak *versus* kinetic energy and emission angle at the normal emission setting of the manipulator. The one-dimensional graph on top shows the integration over the full energy range. (*b*) Polar angle scan 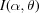 at 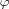 = 47° after background subtraction and peak integration in the energy domain. (*c*) Intensity distribution 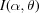 averaged over the 21 measured φ positions. (*d*) Polar scan from panel (*b*) after normalization.

We conclude this section with a brief discussion of the normalization procedure applied to the angle-scanned data in Fig. 9(*c*) ▸. Processing of angle-scanned XPD data from a two-dimensional electron analyser is more complex than from conventional channeltron-based detectors because the measured angle distribution is modified by additional physical and instrumental effects (Greif *et al.*, 2014 ▸). Such effects include the angular dependence of the differential photoionization cross section, the cross section of the illuminated and the analysed volume, as well as angular inhomogeneities of the electron lens and the detector (transmission function). Fig. 11(*a*) ▸ shows the detector image of the N 1*s* peak at the normal emission setting of the manipulator. The distribution of photoelectrons has a pronounced dependence on the polar emission angle α that is extremely sensitive to the distance of the sample from the entrance lens of the analyser. Only after measuring the data presented here we found that the transmission function could be flattened significantly by more careful alignment of the beam, the sample and the focal point of the analyser using a reference sample with well defined emission angles. However, a normalization step is still necessary in any case because the transmission curve is never perfectly flat and because of the polar dependence of the photoemission matrix element.

The normalization procedure is demonstrated on an excerpt from the raw data of the XPD measurement. A more detailed description is given in the supporting information. Fig. 11(*b*) ▸ shows the photoelectron intensity for a single polar scan trace at φ = 47° after peak integration. The image contains diffraction features on top of the slowly varying, non-structural α and θ distribution. The normalization function 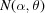 is calculated by averaging 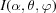 over all measured φ settings and subsequent smoothing. Panel (*c*) shows that the diffraction features are washed out after averaging. This is most easily obtained if the azimuthal scan steps do not coincide with the symmetry of the sample. The normalization function is smoothed in α and θ using a locally weighted regression (LOESS) algorithm (Cleveland *et al.*, 1992 ▸) with a smoothing factor large enough so that the smooth distribution varies slower than the diffraction features. By dividing 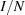 we finally obtain the distribution shown in panel (*d*). The features that are not related to diffraction have been successfully removed. The normalized distribution is finally mapped to the stereographic representation in Fig. 9(*c*) ▸.

### 4.2. Quantum well states in a metal-organic network   

**Figure 12:**
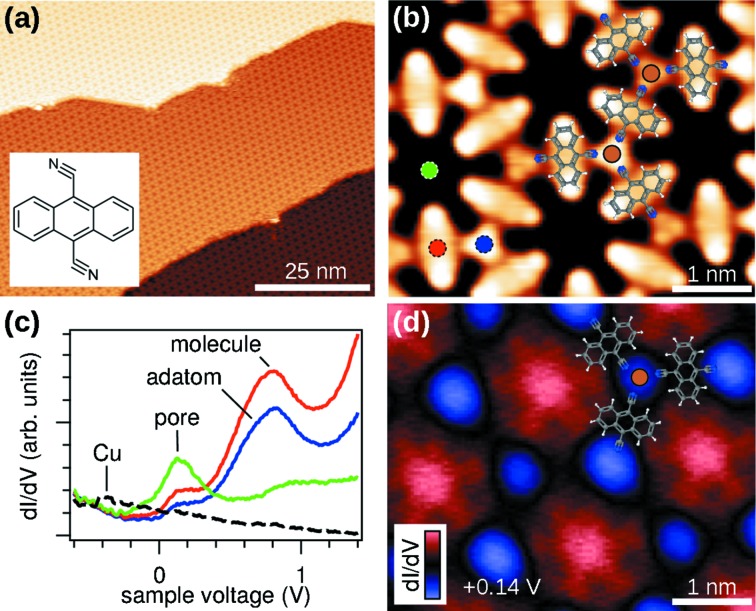
Self-assembled porous network of DCA molecules on Cu(111). (*a*) Wide-area STM topography scan (+2.0 V, 50 pA, 300 K). The inset shows the structural formula of DCA. (*b*) High-resolution STM topography image recorded with a DCA molecule attached to the STM tip (−0.5 V, 100 pA, 4.4 K). An (approximate) model of the molecular structure is overlaid. (*c*) 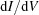 spectra (metal tip, open feedback loop) at the positions marked in panel (*b*) (solid curves), and on clean Cu(111) (dashed curve). Positive voltage corresponds to unoccupied states. (*d*) 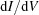 map at +0.14 V showing the lateral distribution of the QWS (metal tip, open feedback loop). The image is slightly slanted due to a small drift during the scan which takes several hours.

Metal-coordinated organic networks provide one possible route to integrate and connect molecular electronic devices with the help of self-assembly (Barth *et al.*, 2005 ▸). In these networks, the spatial extent and the energetic alignment of the electronic states at the interface can be tuned by a judicious choice of molecular building blocks (Scheybal *et al.*, 2009 ▸; Seufert *et al.*, 2013 ▸; Wang *et al.*, 2013 ▸). The mutual interaction of electronic states of adsorbate and substrate is, however, complex and poses a challenge to current numerical methods for theoretical predictions. Experimentally, the properties of occupied electronic states in ordered systems, including their degree of localization, are probed efficiently in ARPES (Lingle Jr *et al.*, 1994 ▸; Lobo-Checa *et al.*, 2009 ▸; Puschnig *et al.*, 2011 ▸). STM and STS probe the local density of states directly, and are able to detect unoccupied states. Both technical features are helpful in the case of a two-dimensional metal-organic network of 9,10-dicyano-anthracene (DCA) molecules (Zhang *et al.*, 2014 ▸). Grown by molecular beam deposition on a clean Cu(111) substrate at room temperature, this network exhibits a long-range periodic 8 × 8 porous superstructure as can be seen in the STM image in Fig. 12(*a*) ▸. The detailed topography image in panel (*b*), measured after attaching a single DCA molecule to the STM tip, shows the arrangement of the molecules and the threefold coordination of the cyano groups with Cu adatoms with submolecular resolution.


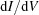
 spectra of the clean and DCA covered Cu(111) surface are shown in Fig. 12(*c*) ▸. The kink in the clean spectrum marks the onset of the Shockley surface state at 0.43 eV below the Fermi level. In strong contrast, the spectra of the DCA network, probed at different sites in the unit mesh, show distinct peaks of unoccupied states. Based on the site dependence of their amplitude we assign the peak at +0.8 eV to the molecular lattice, and the peak at +0.14 eV to a surface quantum well state (QWS) inside the pore (Zhang *et al.*, 2014 ▸). The confined spatial distribution of the QWS peak becomes evident in a constant-height 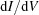 map at +0.14 V bias in panel (*d*).

**Figure 13:**
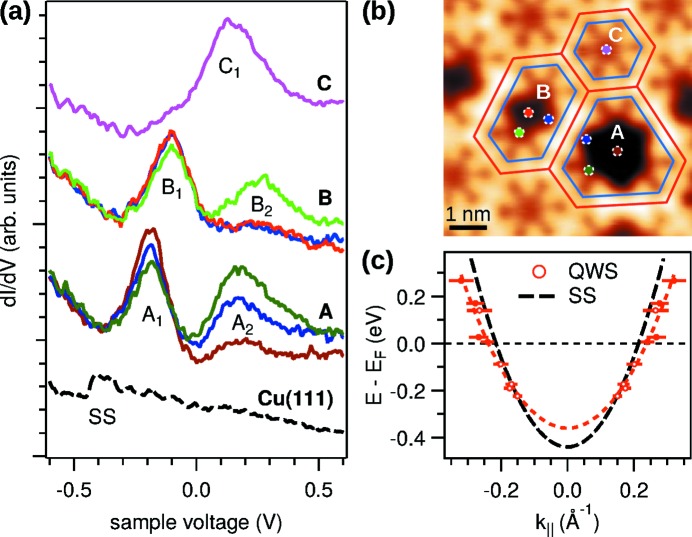
Quantum well states in the pores of the DCA network. (*a*) 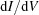 spectra of QWS in pores *A*–*C*. The dashed black curve is a reference spectrum measured on Cu(111), the band bottom of the surface state is labeled SS. (*b*) Topography image of three different sizes of pores at a Y intersection of domain boundaries (−1.0 V, 50 pA, 4.4 K). Blue and red lines, respectively, indicate the boundary of the estimated and maximum area of the quantum well. Dots indicate the probed locations in panel (*a*). (*c*) Energy dispersion of the quantum well states. Red dots are deduced from the peak position and pore size measured in a series of STS and STM measurements. Error bars indicate the uncertainty of determining the size of the pore. The fine dashed curve is a parabolic fit through the data points. The broken curve is the dispersion of the Shockley surface state on bare Cu(111) for reference.

Quantum well states are a result of the confinement of a dispersive state, in this case the free electron-like Shockley surface state of Cu(111), in a potential well imposed by an atomic structure of lower dimensionality (Crommie *et al.*, 1993 ▸; Bürgi *et al.*, 1998 ▸; Baumberger *et al.*, 2002 ▸; Seufert *et al.*, 2013 ▸). The confinement can be treated in the same way as the quantum mechanical particle in a box. The states inside the pore have to fulfill both the quadratic dispersion relation of the surface state, 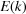 = 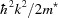, and the boundary conditions of the quantum well which allow only a discrete series of states. The allowable wavevectors 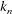 are given essentially by the reciprocal area of the quantum well (Li *et al.*, 1998 ▸; Kaufman *et al.*, 1999 ▸). Since larger pores are present in small concentration at domain boundaries of the DCA network, states with different wavevectors can be probed with STS as illustrated in Fig. 13 ▸. Panel (*a*) shows that the first-order peak in the largest pore *A* appears at a lower energy than the corresponding peak in the smaller pores *B* and *C*. In addition to the first-order peak measured in all pores, second-order peaks are observed in the larger pores *A* and *B*. Using the estimated effective area of the quantum wells [blue boundaries in panel (*b*)], the dispersion of the QWS is plotted in panel (*c*). We notice that, with respect to the unperturbed surface state, the dispersion of the QWS is shifted by 80 meV, and the effective mass is slightly (but not significantly) increased. We interpret the shift as a result of the overlap of the wavefunction with the finite confining barrier imposed by the molecular network (Zhang *et al.*, 2014 ▸). This way, QWS can be used as a sensitive probe of the potential landscape in molecular adsorbate systems.

### 4.3. Circular dichroism in photoelectron diffraction   

A bending magnet produces a superposition of linearly and circularly polarized synchrotron radiation. In the deflection plane, the light is linearly polarized, whereas the light emitted out of the plane contains a significant fraction of circularly polarized light. At the PEARL beamline, the trajectory of the stored electron beam inside the bending magnet can be tilted to extract partially polarized light in the same way as introduced earlier at the PolLux beamline X07DA (Raabe *et al.*, 2008 ▸; Dunn *et al.*, 2004 ▸).

**Figure 14:**
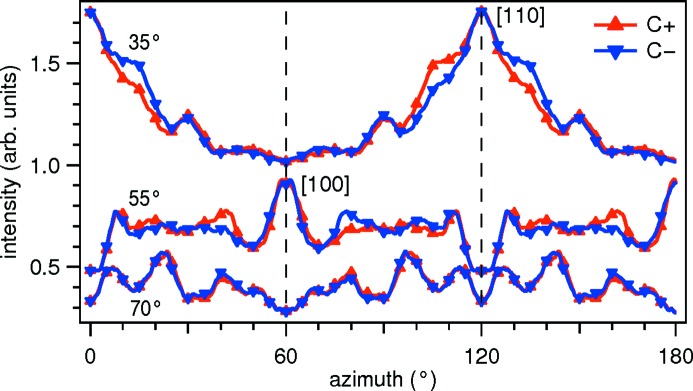
Angle-scanned photoelectron diffraction of the Cu 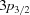 photoelectron peak of a Cu(111) surface measured with left- (C−) and right-handed (C+) circularly polarized light at 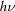 = 679.5 eV and 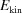 = 600 eV. Three pairs of azimuthal distribution curves are measured at polar angles of 35°, 55° and 70°, cutting across the [110], the [100] and an arbitrary crystal direction, respectively. The curves are symmetrized for a slight difference in the degree of polarization at the two beamline settings. Sparse markers are drawn at every tenth data point.

Circularly polarized radiation is often used to study ordered magnetic moments in atomic systems due to X-ray magnetic circular dichroism. Furthermore, circular dichroism in the angular distribution of photoelectrons has also been observed for non-magnetic systems. In Fig. 14 ▸, we demonstrate the transfer of angular momentum from the circularly polarized photon to the emitted photoelectron, which gives rise to a parallax shift of the forward-focusing peak in the angular distribution of the photoelectron intensity (Daimon, 2001 ▸). The curves show the integrated area of the Cu 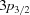 photoelectron peak as a function of the azimuthal rotation angle φ (*cf*. Fig. 4 ▸) at three specific polar angles θ. In the scan at 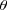 = 35°, the peak and shoulder pattern at 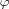 = 120° is attributed to the forward-focusing of the photoelectron along the [110] direction (the nearest-neighbor direction in the Cu f.c.c. crystal). While the photoelectrons excited by linearly polarized photons are detected exactly at 120°, the photoelectrons which were excited by a circularly polarized photon deviate from the straight path and give rise to the shoulders at either side of the [110] direction, depending on the helicity of the photon. The effect is also seen in the [100] direction which corresponds to the second-nearest neighbor direction. Since they depend on the distance between the emitting and the scattering atom, such forward-focusing parallax shifts can be used as a means to measure interatomic distance (Daimon, 2001 ▸). If the atomic geometry is known, the variation of the forward focusing peak intensity can reveal site-specific local electronic and magnetic information (Matsui *et al.*, 2008 ▸, 2015 ▸).

## 5. Summary   

The performance measurements and the scientific examples show that the PEARL beamline of the Swiss Light Source is equipped for a wide range of surface science problems which can benefit from a combination of complementary experimental techniques. In particular, atomic structure can be studied with both local and space-averaging techniques. PEARL is one of very few synchrotron beamlines world-wide that are dedicated to photoelectron diffraction in angle- and energy-scanned modes. Because the instrumentation is mostly the same as for most photoemission spectroscopy methods, the beamline also supports the spectroscopy of core levels, resonant excitations, Auger modes or valence bands. The high resolution and stable imaging over several hours demonstrate the successful implementation of a low-temperature scanning tunneling microscope at a synchrotron facility. PEARL is open to users from the surface science community. Proposals are accepted semi-annually during the regular calls of the Swiss Light Source.

## Supplementary Material

Supporting sections. Section S1: Energy resolution. Section S2: Data processing of hemispherical scans. DOI: 10.1107/S1600577516018646/ve5059sup1.pdf

## Figures and Tables

**Table 1:** Specifications of the X-ray optics

Source type	Bending magnet
Mirrors	2 × toroidal
Monochromator	Planar grating, uncollimated beam
Gratings	600 and 1200 lines mm^−1^
Energy range	60–2000 eV
Beam size (collimated)	190 µm × 70 µm
Beam size (uncollimated)	1100 µm × 1300 µm
Maximum flux	2 × 10^11^ photons s^−1^
Photon energy at maximum flux	800 eV
Ultimate energy resolution ( 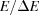 )	7000
Polarization modes	Linear horizontal, elliptical (left/right)

**Table 2:** Specifications of the surface preparation system

Base pressure	4 × 10^−10^ mbar
Sample cleaning	Ar ion sputtering
Heating, radiative	1200 K (100 W)
Heating, direct current	12 A, 60 V
Heating, e-beam	1500 K, under construction
Temperature measurement, infrared	625–1575 K
Cooling	40 K (LHe), 77 K (LN2)
Organic evaporator	Six crucibles, up to 900 K
Gated user ports	2 × DN40CF
LEED/Auger	Omicron SpectaLEED
Residual gas analysis	0–200 a.m.u.
Gas dosing	Leak valve
Vapor deposition	Leak valve
Load lock	1 × 10^−7^ mbar
Sample transfer	Four spaces
Sample storage	22 spaces (2 × 10^−10^ mbar)

**Table 3:** Specifications of the scanning probe microscopy system

Base pressure	1 × 10^−10^ mbar
STM	Omicron LT-STM, Matrix electronics
Detection	Tunneling current
Operating temperature	4.2, 77, 298 K
Gas dosing	Xe/CO *in situ*

**Table 4:** Specifications of the photoelectron spectroscopy system

Base pressure	2 × 10^−10^ mbar
Detector type	Photoelectron spectrometer
Detector model	Scienta EW4000
Energy resolution 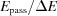	1750
Angle resolution	0.5°
Manipulator	Carving 2.0
Goniometer	Three translations, three rotations
Polar rotation	0° to 180° (60° = normal incidence)
Tilt rotation	−28° to +28°
Azimuthal rotation	−180° to +180°
Cooling	35 K
Heating, radiative	400 K
Sample mounting	Omicron-style sample plate
Photon flux monitoring	Photocurrent on Pt mirror
	Photocurrent on Au mesh

**Table 5:** Summary of measured performance values of the beamline optics Maximum photon flux is measured at *h*ν = 800 eV, energy resolution at 400 eV. G 600 (G 1200) denotes the 600 lines mm^−1^ (1200 lines mm^−1^) grating. See text and supporting information for details

Property	Unit	G 600	G 1200
Maximum photon flux	photons s^−1^	1.7 × 10^11^	5.2 × 10^10^
Photon flux (small aperture)	photons s^−1^	5.6 × 10^9^	3.7 × 10^9^
Resolving power (XAS)		5550	6860
Energy resolution (XAS)	meV	72	58
Energy resolution (XPS)	meV	103	98

## References

[bb1] Auwärter, W., Kreutz, T. J., Greber, T. & Osterwalder, J. (1999). *Surf. Sci.* **429**, 229–236.

[bb2] Auwärter, W., Muntwiler, M., Osterwalder, J. & Greber, T. (2003). *Surf. Sci.* **545**, L735–L740.

[bb3] Barth, J. V. (2007). *Annu. Rev. Phys. Chem.* **58**, 375–407. 10.1146/annurev.physchem.56.092503.141259 17430091

[bb4] Barth, J. V., Costantini, G. & Kern, K. (2005). *Nature (London)*, **437**, 671–679. 10.1038/nature04166 16193042

[bb5] Baumberger, F., Greber, T., Delley, B. & Osterwalder, J. (2002). *Phys. Rev. Lett.* **88**, 237601. 10.1103/PhysRevLett.88.237601 12059398

[bb6] Berthold, W., Rebentrost, F., Feulner, P. & Höfer, U. (2004). *Appl. Phys. Mater. Sci. Process.* **78**, 131–140.

[bb7] Booth, N. A., Davis, R., Toomes, R., Woodruff, D. P., Hirschmugl, C., Schindler, K.-M., Schaff, O., Fernandez, V., Theobald, A., Hofmann, P., Lindsay, R., Gießel, T., Baumgärtel, P. & Bradshaw, A. M. (1997). *Surf. Sci.* **387**, 152–159.

[bb8] Bürgi, L., Jeandupeux, O., Hirstein, A., Brune, H. & Kern, K. (1998). *Phys. Rev. Lett.* **81**, 5370–5373.

[bb9] Chen, C. T. & Sette, F. (1989). *Rev. Sci. Instrum.* **60**, 1616–1621.

[bb10] Cleveland, W. S., Grosse, E. & Shyu, M.-J. (1992). *Software for locally weighted regression*, http://www.netlib.org/a/dloess.

[bb11] Corso, M., Verstraete, M. J., Schiller, F., Ormaza, M., Fernández, L., Greber, T., Torrent, M., Rubio, A. & Ortega, J. E. (2010). *Phys. Rev. Lett.* **105**, 016101. 10.1103/PhysRevLett.105.016101 20867467

[bb12] Crommie, M. F., Lutz, C. P. & Eigler, D. M. (1993). *Science*, **262**, 218–220. 10.1126/science.262.5131.218 17841867

[bb13] Daimon, H. (2001). *Phys. Rev. Lett.* **86**, 2034–2037. 10.1103/PhysRevLett.86.2034 11289848

[bb14] Despont, L., Koitzsch, C., Clerc, F., Garnier, M. G., Aebi, P., Lichtensteiger, C., Triscone, J.-M., Garcia de Abajo, F. J., Bousquet, E. & Ghosez, P. (2006). *Phys. Rev. B*, **73**, 094110.

[bb15] Duncan, D. A., Choi, J. I. J. & Woodruff, D. P. (2012). *Surf. Sci.* **606**, 278–284.

[bb16] Dunn, J. H. (2004). *AIP Conf. Proc.* **705**, 65–68.

[bb17] Fadley, C. S. (1984). *Prog. Surf. Sci.* **16**, 275–388.

[bb18] Fadley, C. S. (2010). *J. Electron Spectrosc. Relat. Phenom.* **178–179**, 2–32.

[bb19] Fasel, R., Aebi, P., Agostino, R. G., Naumović, D., Osterwalder, J., Santaniello, A. & Schlapbach, L. (1996). *Phys. Rev. Lett.* **76**, 4733–4736. 10.1103/PhysRevLett.76.4733 10061367

[bb20] Fasel, R., Wider, J., Quitmann, C., Ernst, K.-H. & Greber, T. (2004). *Angew. Chem. Int. Ed.* **43**, 2853–2856. 10.1002/anie.200353311 15150767

[bb21] Föhlisch, A., Feulner, P., Hennies, F., Fink, A., Menzel, D., Sanchez-Portal, D., Echenique, P. M. & Wurth, W. (2005). *Nature (London)*, **436**, 373–376. 10.1038/nature03833 16034414

[bb22] Gamou, Y., Terai, M., Nagashima, A. & Oshima, C. (1997). *Sci. Rep. Res. Inst. Tohoku Univ.* A**44**, 211–214.

[bb23] García de Abajo, F. J., Van Hove, M. A. & Fadley, C. S. (2001). *Phys. Rev. B*, **63**, 075404.

[bb24] Grad, G. B., Blaha, P., Schwarz, K., Auwärter, W. & Greber, T. (2003). *Phys. Rev. B*, **68**, 085404.

[bb25] Greber, T., Šljivančanin, Ž., Schillinger, R., Wider, J. & Hammer, B. (2006). *Phys. Rev. Lett.* **96**, 056103. 10.1103/PhysRevLett.96.056103 16486958

[bb26] Greif, M., Castiglioni, L., Becker-Koch, D., Osterwalder, J. & Hengsberger, M. (2014). *J. Electron Spectrosc. Relat. Phenom.* **197**, 30–36.

[bb27] Jaouen, T., Razzoli, E., Didiot, C., Monney, G., Hildebrand, B., Vanini, F., Muntwiler, M. & Aebi, P. (2015). *Phys. Rev. B*, **91**, 161410.

[bb28] Kato, M., Morishita, Y., Oura, M., Yamaoka, H., Tamenori, Y., Okada, K., Matsudo, T., Gejo, T., Suzuki, I. & Saito, N. (2007). *J. Electron Spectrosc. Relat. Phenom.* **160**, 39–48.

[bb29] Kaufman, D. L. (1999). *Am. J. Phys.* **67**, 133–141.

[bb30] Kreutz, T. J., Greber, T., Aebi, P. & Osterwalder, J. (1998). *Phys. Rev. B*, **58**, 1300–1317.

[bb31] Li, J., Schneider, W.-D., Berndt, R. & Crampin, S. (1998). *Phys. Rev. Lett.* **80**, 3332–3335.

[bb32] Lingle, R. L. Jr, Padowitz, D. F., Jordan, R. E., McNeill, J. D. & Harris, C. B. (1994). *Phys. Rev. Lett.* **72**, 2243–2246. 10.1103/PhysRevLett.72.2243 10055825

[bb33] Lobo-Checa, J., Matena, M., Müller, K., Dil, J. H., Meier, F., Gade, L. H., Jung, T. A. & Stöhr, M. (2009). *Science*, **325**, 300–303. 10.1126/science.1175141 19608913

[bb34] Matsui, F., Fujita, M., Ohta, T., Maejima, N., Matsui, H., Nishikawa, H., Matsushita, T. & Daimon, H. (2015). *Phys. Rev. Lett.* **114**, 015501. 10.1103/PhysRevLett.114.015501 25615477

[bb35] Matsui, F., Matsushita, T., Kato, Y., Hashimoto, M., Inaji, K., Guo, F. Z. & Daimon, H. (2008). *Phys. Rev. Lett.* **100**, 207201. 10.1103/PhysRevLett.100.207201 18518574

[bb36] Meyer, M., Stähler, J., Kusmierek, D. O., Wolf, M. & Bovensiepen, U. (2008). *Phys. Chem. Chem. Phys.* **10**, 4932–4938. 10.1039/b807314g 18688537

[bb37] Morscher, M., Nolting, F., Brugger, T. & Greber, T. (2011). *Phys. Rev. B*, **84**, 140406.

[bb38] Muntwiler, M., Auwärter, W., Baumberger, F., Hoesch, M., Greber, T. & Osterwalder, J. (2001). *Surf. Sci.* **472**, 125–132.

[bb39] Muntwiler, M., Auwärter, W., Seitsonen, A. P., Osterwalder, J. & Greber, T. (2005). *Phys. Rev. B*, **71**, 121402.

[bb40] Oberta, P., Flechsig, U., Muntwiler, M. & Quitmann, C. (2011). *Nucl. Instrum. Methods Phys. Res. A*, **635**, 116–120.

[bb41] Osterwalder, J., Aebi, P., Fasel, R., Naumović, D., Schwaller, P., Kreutz, T., Schlapbach, L., Abukawa, T. & Kono, S. (1995). *Surf. Sci.* **331–333**, 1002–1014.

[bb42] Pawlak, R., Fremy, S., Kawai, S., Glatzel, T., Fang, H., Fendt, L.-A., Diederich, F. & Meyer, E. (2012). *ACS Nano*, **6**, 6318–6324. 10.1021/nn301774d 22659024

[bb43] Pawlak, R., Marot, L., Sadeghi, A., Kawai, S., Glatzel, T., Reimann, P., Goedecker, S., Güntherodt, H.-J. & Meyer, E. (2015). *Sci. Rep.* **5**, 13143. 10.1038/srep13143 PMC4542518 26268430

[bb44] Petersen, H., Jung, C., Hellwig, C., Peatman, W. B. & Gudat, W. (1995). *Rev. Sci. Instrum.* **66**, 1–14.

[bb45] Piamonteze, C., Flechsig, U., Rusponi, S., Dreiser, J., Heidler, J., Schmidt, M., Wetter, R., Calvi, M., Schmidt, T., Pruchova, H., Krempasky, J., Quitmann, C., Brune, H. & Nolting, F. (2012). *J. Synchrotron Rad.* **19**, 661–674. 10.1107/S0909049512027847 22898943

[bb46] Puschnig, P., Reinisch, E.-M., Ules, T., Koller, G., Soubatch, S., Ostler, M., Romaner, L., Tautz, F. S., Ambrosch-Draxl, C. & Ramsey, M. G. (2011). *Phys. Rev. B*, **84**, 235427.

[bb47] Raabe, J., Tzvetkov, G., Flechsig, U., Böge, M., Jaggi, A., Sarafimov, B., Vernooij, M. G. C., Huthwelker, T., Ade, H., Kilcoyne, D., Tyliszczak, T., Fink, R. H. & Quitmann, C. (2008). *Rev. Sci. Instrum.* **79**, 113704. 10.1063/1.3021472 19045892

[bb48] Reinert, F., Nicolay, G., Schmidt, S., Ehm, D. & Hüfner, S. (2001). *Phys. Rev. B*, **63**, 115415.

[bb49] Scheybal, A., Müller, K., Bertschinger, R., Wahl, M., Bendounan, A., Aebi, P. & Jung, T. A. (2009). *Phys. Rev. B*, **79**, 115406.

[bb50] Scheybal, A., Ramsvik, T., Bertschinger, R., Putero, M., Nolting, F. & Jung, T. (2005). *Chem. Phys. Lett.* **411**, 214–220.

[bb51] Schillinger, R., Šljivančanin, Ž., Hammer, B. & Greber, T. (2007). *Phys. Rev. Lett.* **98**, 136102. 10.1103/PhysRevLett.98.136102 17501218

[bb52] Seufert, K., Auwärter, W., García de Abajo, F. J., Ecija, D., Vijayaraghavan, S., Joshi, S. & Barth, J. V. (2013). *Nano Lett.* **13**, 6130–6135. 10.1021/nl403459m 24245663

[bb53] Strocov, V. N., Schmitt, T., Flechsig, U., Schmidt, T., Imhof, A., Chen, Q., Raabe, J., Betemps, R., Zimoch, D., Krempasky, J., Wang, X., Grioni, M., Piazzalunga, A. & Patthey, L. (2010). *J. Synchrotron Rad.* **17**, 631–643. 10.1107/S0909049510019862 PMC2927903 20724785

[bb54] Treier, M., Ruffieux, P., Fasel, R., Nolting, F., Yang, S., Dunsch, L. & Greber, T. (2009). *Phys. Rev. B*, **80**, 081403.

[bb55] Wang, S., Wang, W., Tan, L. Z., Li, X. G., Shi, Z., Kuang, G., Liu, P. N., Louie, S. G. & Lin, N. (2013). *Phys. Rev. B*, **88**, 245430.

[bb56] Westerström, R., Dreiser, J., Piamonteze, C., Muntwiler, M., Weyeneth, S., Brune, H., Rusponi, S., Nolting, F., Popov, A., Yang, S., Dunsch, L. & Greber, T. (2012). *J. Am. Chem. Soc.* **134**, 9840–9843. 10.1021/ja301044p 22582902

[bb57] Westerström, R., Dreiser, J., Piamonteze, C., Muntwiler, M., Weyeneth, S., Krämer, K., Liu, S.-X., Decurtins, S., Popov, A., Yang, S., Dunsch, L. & Greber, T. (2014). *Phys. Rev. B*, **89**, 060406.

[bb58] Wider, J., Greber, T., Wetli, E., Kreutz, T. J., Schwaller, P. & Osterwalder, J. (1998). *Surf. Sci.* **417**, 301–310.

[bb59] Woodruff, D. P. (2007). *Surf. Sci. Rep.* **62**, 1–38.

[bb60] Zhang, J., Shchyrba, A., Nowakowska, S., Meyer, E., Jung, T. A. & Muntwiler, M. (2014). *Chem. Commun.* **50**, 12289–12292. 10.1039/c4cc03941f 25180248

