# Supplementary material for: Surface science at the PEARL beamline of the Swiss Light Source
Source: J Synchrotron Radiat. 2017 Jan 1;24(Pt 1):354–66. doi: 10.1107/S1600577516018646 (PMC5182030; doi:10.1107/S1600577516018646)
Supplement: Supplementary file 1 [file s-24-00354-sup1.pdf]

JOURNAL OF SYNCHROTRON RADIATION

Volume 24 (2017)

Supporting information for article:

Surface science at the PEARL beamline of the Swiss Light Source

Matthias Muntwiler, Jun Zhang, Roland Stania, Fumihiko Matsui, Peter Oberta, Uwe Flechsig, Luc Patthey, Christoph Quitmann, Thilo Glatzel, Roland Widmer, Ernst Meyer, Thomas A. Jung, Philipp Aebi, Roman Fasel and Thomas Greber

Supporting Information to:
Surface Science at the PEARL Beamline of the Swiss Light Source

Matthias Muntwiler[a], Jun Zhang[a], Roland Stania[a,d], Fumihiko Matsui[f], Peter Oberta[a,g], Uwe Flechsig[a], Luc Patthey[a], Christoph Quitmann[a,h], Thilo Glatzel[b], Roland Widmer[e], Ernst Meyer[b], Thomas A. Jung[a,b], Philipp Aebi[c], Roman Fasel[e], and Thomas Greber[d]

[a]*Paul Scherrer Institut, Villigen, Switzerland*
[b]*Universität Basel, Basel, Switzerland*
[c]*Université de Fribourg, Fribourg, Switzerland*
[d]*Universität Zürich, Zürich, Switzerland*
[e]*Swiss Federal Laboratories for Materials Science and Technology (Empa), Dübendorf, Switzerland*
[f]*Nara Institute of Science and Technology (NAIST), Nara, Japan*
[g]*Institute of Physics, Academy of Sciences of the Czech Republic, Praha, Czech Republic*
[h]*MAX IV Laboratory, Lund University, Lund, Sweden*

1 Energy Resolution

1.1 Absorption Spectra

For the gas phase spectra of nitrogen, the ion yield is measured in a gas cell installed after the exit slit of the monochromator. The gas pressure in the cell is set to 2×10^{-2} mbar, and total ion yield is measured using a current-to-voltage amplifier at a gain setting of 10^8 V/A and a bias of 45 V. The spectra are recorded in the *flying* mode of the monochromator, i.e. the mirror and grating are moving at constant speed while the current energy and the signal are recorded at a frequency of 10 Hz.

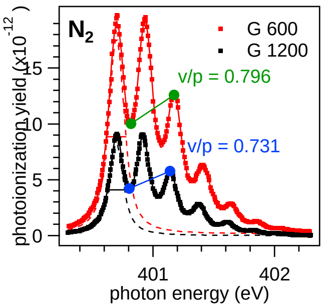

Figure 1: Measured total yield absorption spectra of nitrogen for the 600 lines/mm grating (G600) and the 1200 lines/mm grating (G1200). The solid lines are least-squares fits of Voigt functions as described in the text. Full width at half maximum (FWHM) of the lowest-energy peaks (dashed line) is indicated by horizontal bars. The intensity ratio between the first valley and third peak (v/p) is indicated.

Grating 1/mm	Full Peak meV	Voigt Fit free Lorentzian meV	Gaussian meV	Voigt Fit constrained Lorentzian meV	Gaussian meV
600	150	132.2	49.0	113	76.9
1200	135	124.5	24.8	113	49.6

Table 1: Summary of gas phase x-ray absorption measurements of nitrogen. The *full peak* column shows the total FWHM of the principal peak of each spectrum. The *Voigt fit* columns show the resulting FWHM of the Lorentzian and Gaussian components from a *free*, and a *constrained* curve fit. The values are full width at half maximum (FWHM).

Grating 1/mm	Peak Width	Voigt Fit free	Voigt Fit constrained	Peak/Valley	Ray Tracing
600	5379	8174	5210	5549	5500
1200	7352	16142	8076	6858	7000

Table 2: Resolving power $E/\Delta E$ calculated according to the different analysis methods explained in the text. The values of the *peak fit* column are estimated using Eq. 1. The theoretical ray tracing results are from [1].

There are several ways to analyse the spectra shown in Fig. 1 and to deduce the instrumental broadening. The results are summarized in Tables 1 and 2. Table 1 lists ΔE values as absolute full width at half maximum (FWHM). Table 2 lists the corresponding resolving power $E/\Delta E$.

The nitrogen spectra are fit with model spectra consisting of the sum of seven Voigt profiles in a Levenberg-Marquardt least-square curve fitting algorithm. A Voigt profile is the convolution of a Lorentzian and a Gaussian profile, where the Lorentzian component models the natural line shape, and the Gaussian component the broadening due to finite instrumental resolution. If the natural line width Γ is known, the width ΔE of the Gaussian component can be estimated from the FWHM w of the peak according to

$$\Delta E = w\sqrt{1 - \frac{\Gamma}{w}}. \tag{1}$$

Because the widths of the two components are correlated, it is difficult to determine both at the same time. As can be seen in the *free Voigt fit* columns of Table 1, the resulting Lorentzian widths for nitrogen are significantly larger than the smallest reported literature value 113 meV [2]. This results in an underestimated instrumental broadening (Gaussian component ΔE) compared to the ray tracing results in Table 2. Thus, for a conservative estimate of the instrumental energy resolution, we constrain the lifetime parameter Γ to 113 meV (*constrained Voigt fit* columns).

The Valley-to-Peak ratio, i.e. the intensity at the minimum of the first valley divided by the intensity of the third peak (cf. Fig. 1), is another sensitive measure of the energy resolution [3]. The advantage is that it is independent of curve fitting and the calibration of the energy scale. The measured v/p values are evaluated by comparison to calculated model spectra using a same series of Voigt profiles where literature values are inserted for the peak separation and the natural line width parameters [2]. The results are listed in the *peak/valley* column of Table 2. They confirm the theoretical resolving power of ≈ 5500 and ≈ 7000 for the 600 and the 1200 lines/mm gratings, respectively.

1.2 Fermi Edge

The measured width of the Fermi edge of a metallic sample contains the intrinsic thermal broadening in the material and the instrumental broadening by the analyser and the beamline [4]. For small amounts of broadening, these contributions add up quadratically. The instrumental energy resolution

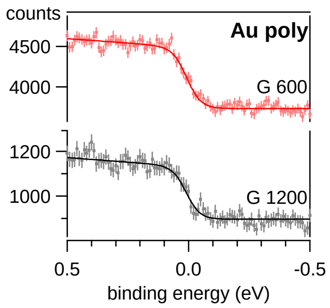

Figure 2: Photoelectron spectra at the Fermi edge of a gold polycrystal measured at $h\nu = 400\,\text{eV}$. The spectra were measured using the 600 lines/mm (G600) or 1200 lines/mm (G1200) monochromator grating as indicated. Dots are electron counts integrated over the 60° acceptance angle of the analyser, and error bars indicate the estimated standard deviation according to Poisson statistics. Lines are curve fits of a Fermi function assuming, to first order, a linear increase of the density of states below E_F.

$h\nu$ eV	Grating 1/mm	T_{FD} K	ΔE meV	$E/\Delta E$	ΔE_A meV	I_s pA
400	600	341 ± 40	103 ± 12	3878 ± 455	73 ± 24	104.5
400	1200	325 ± 62	98 ± 19	4075 ± 783	80 ± 27	50.0
867	600	682 ± 21	207 ± 6	4187 ± 130	75 ± 67	206.5
867	1200	576 ± 33	175 ± 10	4960 ± 281	69 ± 62	46.8

Table 3: Summary of the Fermi edge measurements on polycrystalline gold. T_{FD} is the temperature of the Fermi-Dirac distribution and ΔE the FWHM of the instrumental resolution. ΔE includes the contributions of the beamline optics (Table 2) and of the electron analyser ΔE_A under the measurement conditions described in the text. I_s is the total electron yield.

ΔE expressed as full width at half maximum (FWHM) is then

$$\Delta E^2 = 3.53^2 (T_{FD}^2 - T_s^2) k_B^2, \tag{2}$$

where T_{FD} is the measured temperature parameter of the Fermi-Dirac distribution and T_s the actual temperature of the sample. The factor 3.53 converts temperature to the FWHM of a Gaussian so that the convolution of the Gaussian with a Heaviside function approximates the Fermi-Dirac distribution.

The sample is the surface of polycrystalline gold which is cleaned in situ by ion bombardment. During the measurement, the sample is cooled to $T_s = 40\,\text{K}$ to eliminate the contribution of thermal excitations. The beamline settings are the same as in the gas phase absorption spectra except that the front end aperture is widened to $(240\,\mu\text{rad})^2$. The effect of the wider aperture on the energy resolution is less than 5% as verified in a separate gas phase measurement. The measured spectra and the corresponding Fermi-Dirac curve fits are displayed in Fig. 2. Table 3 shows the resulting energy resolution values calculated according to Eq. 2. Given the energy resolution values ΔE_{opt} of the beamline optics concluded in Sec. 1.1 (ray tracing values from Table 2) we can also calculate the energy resolution

$$\Delta E_A^2 = \Delta E^2 - \Delta E_{opt}^2 \tag{3}$$

of the analyser. The mean value $(76 \pm 17)\,\text{meV}$ from the four measurements is about a factor 3 larger than the nominal value 28 meV calculated from the selected entrance slit (0.2 mm) and the pass energy (50 eV). Though the resolution of the analyser could be improved by lowering the pass energy, the low count rate due to the high-resolution setting of the beamline and the very low photoemission cross-section of the valence band in the soft x-ray regime did not allow so as the acquisition of each displayed spectrum took about 12 hours. Though the photocurrent measurements in Table 3 show a reasonable total electron yield, it has to be noted that most of this current comes from core level excitations whose photoelectron and Auger peaks produce much larger signals.

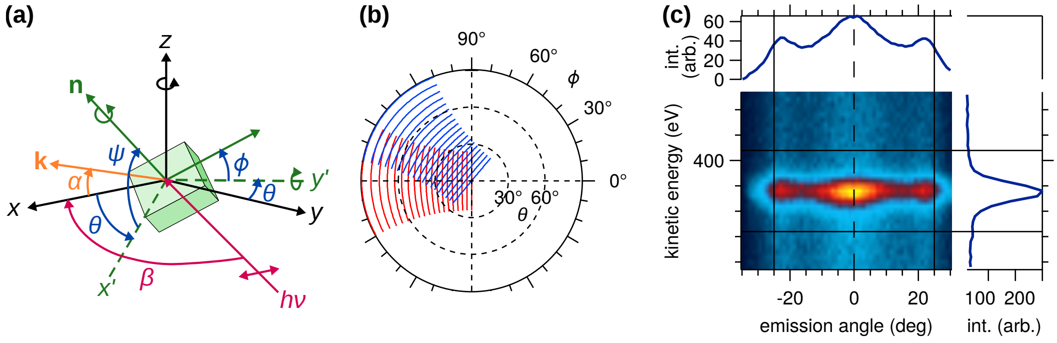

Figure 3: (a) Measurement geometry (see main article). Electrons emitted under the emission angle $-30° < \alpha < +30°$ in the xz plane are detected in parallel. (b) Hemispherical scanning scheme of XPD (see main article). A full scan of emission angles in the hemisphere is a combination of polar (θ) and azimuthal (ϕ) scans. Each of the curved lines in the plot corresponds to the α interval detected in one shot. For clarity, only a few angles are shown. (c) Measured photoelectron intensity of the N 1*s* peak of *h*-BN/Ni(111) versus kinetic energy and emission angle at the normal emission setting of the manipulator. The one-dimensional lines are profiles integrated over the full energy or angle range. Vertical solid lines delimit the angular interval used in the process. Horizontal solid lines show the border of the peak integration interval.

2 Data Processing of Hemispherical Scans

For physical and technical reasons, processing of angle-scanned XPD data from a two-dimensional electron analyser is more complex than from conventional channeltron based detectors [5]. Considering the—in many cases—intuitive interpretation of stereographic diffraction plots [6], we have developed a procedure to generate the same kind of plots from two-dimensional data. In this section, we discuss the detailed data processing in the case of the *h*-BN/Ni(111) system presented in the main article. The computer code is available on-line [7].

A basic dataset of the N 1*s* peak measured with the Scienta EW4000 is shown in Fig. 3(c). The distribution of photoelectron counts has a pronounced dependence on the polar emission angle α. On the one hand, this is due to the angular dependence of the differential photoionization cross-section which for an *s* ground state is proportional to $\cos^2 \alpha$. On the other hand, we notice three maxima that we attribute to inhomogeneous transmission of the electron lens. Other effects, such as the cross-section of the illuminated and the analysed volume or the escape depth may play an additional role. In the case of an atomically thin layer they should, however, be negligible. Thus, for the interpretation of the data, at least the effect of the transmission function needs to be compensated. Since it is currently not possible to separate it from the other effects, we deduce the normalization function from the measured data by averaging and smoothing.

As described in the main text, XPD data is acquired in a two-dimensional manipulator scan of (θ, ϕ) while the analyser measures the intensity distribution $I(E, \alpha)$ at each scan position. In the example, we measure a total of 2184 angle settings[1]. ϕ is varied in steps of 15° spanning the full range of 360°. θ is scanned in steps of 1° from normal emission to 90° grazing emission. Wider ϕ steps can be chosen if their integer multiples do not coincide with the symmetry of the sample, which could otherwise introduce problems in the normalization procedure. The counting time (in *swept* mode) at each angle setting is 10.6 s.

The normalization procedure is shown schematically in Fig. 4. The raw data $I(E, \alpha, \theta, \phi)$ is four-dimensional (a). In the first processing step, the energy axis is eliminated by line-by-line background subtraction and peak integration. This results in a three-dimensional distribution $I(\alpha, \theta, \phi)$ which we display as a two-dimensional strip where one dimension is a flattened manipulator scan, and the other is the emission angle (b). The plot resembles a film strip where each "frame" contains one θ scan at a particular ϕ. The data shows diffraction-related features on top of a slowly varying background.

[1]Only 1820 angles are actually used in the analysis due to a glitch in two of the polar scans.

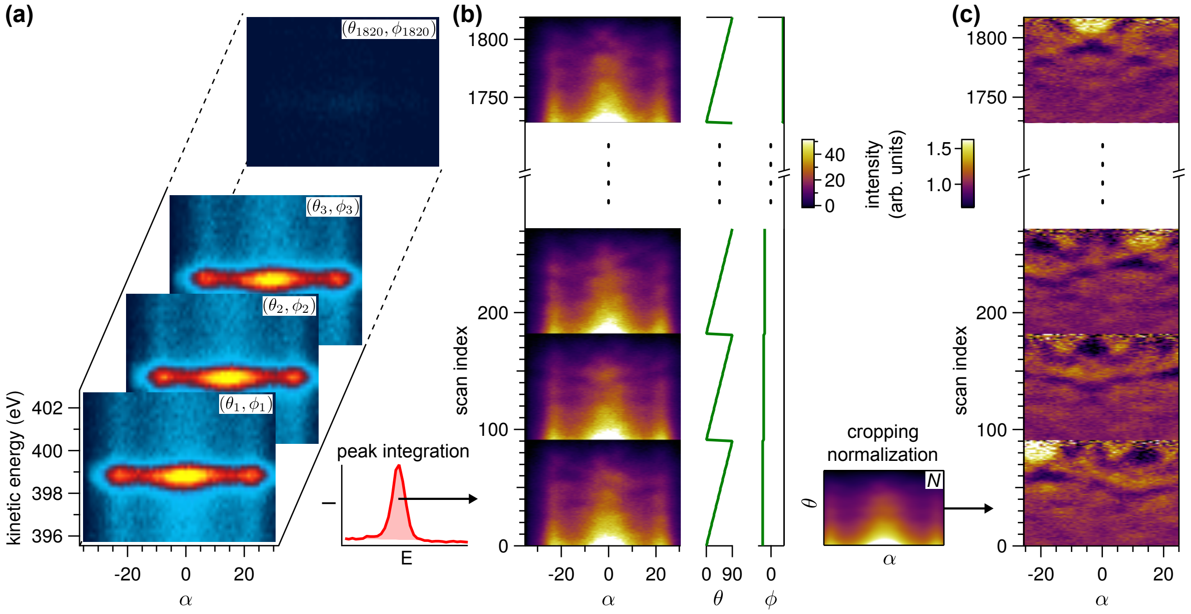

Figure 4: Normalization procedure of angle-scanned XPD data of h-BN/Ni(111). (a) At each angle setting (θ, ϕ) of the manipulator, the analyser acquires a snapshot $I(E, \alpha)$ of the N 1s peak. 1820 angle settings are used in the process. (b) After peak integration, the intensity $I(\theta, \phi, \alpha)$ is plotted versus α and a flat index of the manipulator angle. The actual θ and ϕ values are indicated in the line graphs on the right-hand side. θ is the fast and *phi* the slow scan axis. (c) Normalized data. The smooth normalization function $N(\theta, \alpha)$ is plotted on the lower left.

In the second step, we normalize the data to remove the angular transmission of the analyser, and to get a flat intensity distribution which contains only diffraction-related features. The normalization function $N(\alpha, \theta)$ is computed by averaging over all measured ϕ settings and subsequent smoothing. For smoothing we use a locally-weighted regression algorithm [8]. The smoothing factor is set large enough so that the smoothed distribution varies slower than the expected diffraction features. By dividing I/N we obtain the distribution shown in Fig. 4(c).

In the last step, the normalized distribution $I(\theta, \phi, \alpha)$ is mapped to polar coordinates $I(\theta_s, \phi_s)$ in the reference frame of the sample as described in the main text, and added to the coordinate grid used with conventional detectors [6]. The data points of the transformed distribution are added to the nearest points of the destination grid and normalized according to their numeric weight. The data is plotted in the stereographic projection as shown in Fig. 5, either by overlapping dots as in panel (a), or after interpolation to a Cartesian grid as in panel (b).

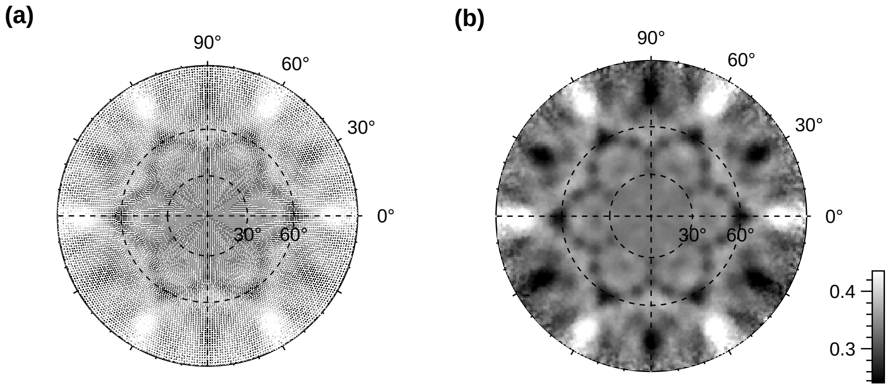

Figure 5: Two representations of the resulting XPD plot. (a) Angular grid consisting of 16376 data points in stereographic projection. The dots are plotted in a small size to make the grid visible. (b) Interpolation to a 181×181 rectangular grid (after projection). The data points are mapped to their nearest-neighbor destination points in the projected coordinate space. The image is smoothed by a 3×3 pixels Gaussian filter. The gray scale is the same in both plots.

References

[1]P. Oberta, et al., Nucl. Instr. and Meth. A **635**, 116 (2011).

[2]M. Kato, et al., J. Electron Spectrosc. Relat. Phenom. **160**, 39 (2007).

[3]C. T. Chen, et al., Rev. Sci. Instrum. **60**, 1616 (1989).

[4]T. J. Kreutz, et al., Phys. Rev. B **58**, 1300 (1998).

[5]M. Greif, et al., J. Electron Spectrosc. Relat. Phenom. **197**, 30 (2014).

[6]J. Osterwalder, et al., Surf. Sci. **331-333**, 1002 (1995).

[7]M. Muntwiler, *PEARL procedures*, `https://gitlab.psi.ch/pearl-public/igor-procs`, 2016.

[8]W. S. Cleveland, et al., *Software for locally-weighted regression*, `http://www.netlib.org/a/dloess`, 1992.
